# Preeminently Robust Neural PPG Denoiser

**DOI:** 10.3390/s22062082

**Published:** 2022-03-08

**Authors:** Ju Hyeok Kwon, So Eui Kim, Na Hye Kim, Eui Chul Lee, Jee Hang Lee

**Affiliations:** 1 Department of AI & Informatics, Graduate School, Sangmyung University, Seoul 03016, Korea; juhuk98@naver.com (J.H.K.); soeui291@gmail.com (S.E.K.); nahelove03@gmail.com (N.H.K.); 2 Department of Human-Centered Artificial Intelligence, Sangmyung University, Seoul 03016, Korea

**Keywords:** photoplethysmography, denoising, universal denoiser

## Abstract

Photoplethysmography (PPG) is a simple and cost-efficient technique that effectively measures cardiovascular response by detecting blood volume changes in a noninvasive manner. A practical challenge in the use of PPGs in real-world applications is noise reduction. PPG signals are likely to be compromised by various types of noise, such as scattering or motion artifacts, and removing such compounding noises using a monotonous method is not easy. To this end, this paper proposes a neural PPG denoiser that can robustly remove multiple types of noise from a PPG signal. By casting the noise reduction problem into a signal restoration approach, we aim to achieve a solid performance in the reduction of different noise types using a single neural denoiser built upon transformer-based deep generative models. Using this proposed method, we conducted the experiments on the noise reduction of a PPG signal synthetically contaminated with five types of noise. Following this, we performed a comparative study using six different noise reduction algorithms, each of which is known to be the best model for each noise. Evaluation results of the peak signal-to-noise ratio (PSNR) show that the neural PPG denoiser is superior in three out of five noise types to the performance of conventional noise reduction algorithms. The salt-and-pepper noise type showed the best performance, with the PSNR of the neural PPG denoiser being 36.6080, and the PSNRs of the other methods were 19.8160 and 32.8234. The Poisson noise type performed the worst, showing a PSNR of 33.0090; the PSNRs of other methods were 35.1822 and 33.4795, respectively. Thereafter, an experiment to recover a signal synthesized with two or more of the five noise types was conducted. When the number of mixed noises was two, three, four, and five, the PSNRs were 29.2759, 27.8759, 26.5608, and 25.9402, respectively. Finally, an experiment to recover motion artifacts was also conducted. The synthesized motion artifact signal was created by synthesizing only a certain ratio of the total signal length. As a result of the motion artifact signal restoration, the PSNRs were 25.2872, 22.8240, 21.2901, and 19.9577 at 30%, 50%, 70%, and 90% motion artifact ratios, respectively. In the three experiments conducted, the neural PPG denoiser showed that various types of noise were effectively removed. This proposal contributes to the universal denoising of continuous PPG signals and can be further expanded to denoise continuous signals in the general domain.

## 1. Introduction

Photoplethysmography (PPG) is a simple and low-cost method to measure blood volume changes by detecting the amount of light reflected by irradiating the skin surface in a noninvasive manner [1]. This technique has received much attention in recent decades as a result of its cost-efficiency and ease of implementation. The technique is thus applied in many ways, notably for convenient monitoring of various basic vital signs, such as pulse, respiration, heart rate variability (HRV), and blood oxygen saturation [1,2,3,4]. However, PPG is vulnerable to noise, and in practical settings, PPG signals measured in the real world are likely to be compromised by various types of noise, including scattering, motion artifacts, and lower blood perfusion [5]. Noise removal plays an important role in the PPG process because noise can distort the shape of a signal.

To this end, there is a growing demand for noise removal techniques to restore PPG signals to their original shape. One line of research has focused on the use of additional sensors to artificially calibrate the observed PPG signal. Thamarai et al. and Biswas et al. proposed a wavelet-based approach to effectively restore the PPG signal from a mixture of signals and multiple noises, especially for the removal of compounded motion noise [6,7,8]. Tanweer et al. introduced an approach that uses an additional sensor to eliminate motion noise in PPG signals. Despite these studies showing relatively competitive performance, it seems onerous because it adopts multiple sensors to necessitate the demanding procedure of consecutive comparisons between the two different signals [9].

Hanyu et al. proposed a lighter denoiser framework without considering additional sensors. Using statistical parameters, they attempted to detect an abnormal pattern of a signal contaminated by motion noise and cut out the detected part afterwards [10]. However, this approach causes irreversible loss of the original PPG signal, which might significantly diminish the quality of the processing outcomes. Hara et al. introduced a hybrid approach using two PPG sensors that captured the optimal parameter of each signal and used them to remove the effect of motion noise afterwards [11]. Despite their relatively high performance, these methods have shortcomings as they are dedicated to a specific type of noise that cannot be applied to other types of noise in general.

The fundamental challenge in denoising PPG signals is that noise is usually generated not only by motion artifacts but also by various types of sporadic noise, owing to signal amplification, electrical interference, and baseline drift. Subsequently, monotonous and fragmented resolutions to the specific types of noise in PPG signals might not be sufficient. For example, wavelet-based or adaptive filter-based methods can successfully remove motion artifacts, but they cannot resolve the denoising problem on high-frequency components owing to sporadic noise generation. They are only effective in restoring the signal shape for a specific section.

Within this context, this paper proposes a neural PPG denoiser (NPD) that can robustly remove multiple types of noise from a PPG signal. By casting the noise reduction problem into a signal restoration approach, we aimed to achieve solid performance in the reduction of different types of noise using a single neural denoiser. Accordingly, a deep neural denoiser built upon transformer-based deep generative models was built, inspired by the super-resolution problem [12], in which a single neural network-based denoiser can learn to cope with diverse types of noise. Experiments showed that the proposed method showed the highest peak signal-to-noise ratio (PSNR) in the removal of five different types of noise (Gaussian, Poisson, salt and pepper, speckle, and uniform noise), compared to (i) that of a wavelet-based denoiser and (ii) that of the best denoiser algorithm for each noise. The contributions of this study are as follows:

- This proposal, NPD, ensures high-quality signal restoration over many different types of noise in comparison to state-of-the-art denoising algorithms.
- The authors suggest a novel computational framework in which transformer-based deep generative models can learn to remove various types of noise in continuous PPG signals by casting the denoising problem into a signal restoration problem.
- This proposal can be expanded to the general signal processing domain as a universal denoiser for continuous signals.

The remainder of this paper is organized as follows. After the introduction in Section 1, the proposal, NPD, and details of the proposal for ways in which deep generative models learn to universally remove various noise types, is outlined in Section 2. This is followed by the experimental settings described in Section 3. The results are presented in Section 4, and the study is concluded with a discussion and suggestions for future work in Section 5.

## 2. Proposed Method

The main goal of this work is to provide a universal denoiser for PPG signals. We note that signals affected by motion artifacts or other noise were considered, and other factors such as blood perfusion were not considered. For this, we adopted the transformer-based deep generative model approach which casts the noise removal of continuous signals to the signal restoration technique. Super-resolution (SR) research in the image-processing domain is a good example [13]. In SR, the central aim is to restore high-resolution images from low-resolution images for many reasons (e.g., distortion, transmission errors, and loss due to compression). The assumption in this study is that PPG signals of low quality (owing to noise) can be equated to low-resolution images in SR settings. If so, the study further assumes that the quality of the PPG signals can be enhanced (i.e., noise removed from the PPG signal) by following the resolution method suggested by the SR to have high-resolution images.

**Figure 1:**
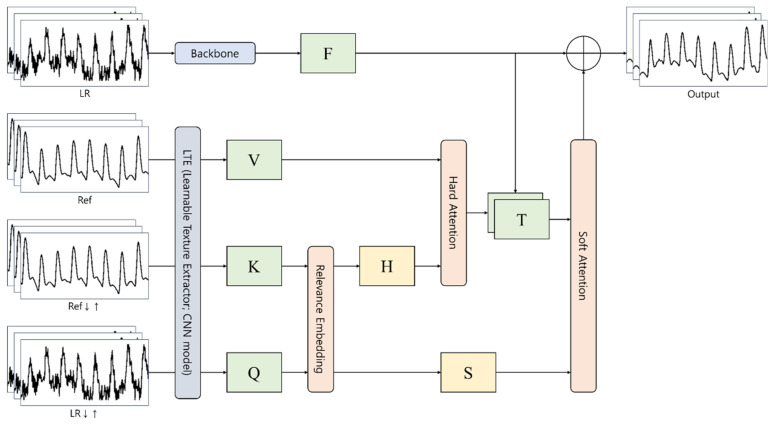
Overall architecture of neural PPG denoiser (NPD). *Q* is the texture features extracted from the upsampled LR image, *K* is the texture features extracted from the upsampled Ref image, after downsampling, *V* is the texture features extracted from the original Ref image, *H* is the hard attention map calculated from the relevance embedding, *S* is the soft attention map calculated from the relevance embedding, *F* is the LR features extracted from the DNN backbone, and *T* is the texture features transmitted to generate the SR output.

This study uses a texture transformer network for image SR (TTSR) as a backbone framework for a PPG denoiser. It is a convolutional neural network (CNN)-based image-restoration technique that incorporates a transformer-based deep generative model [12]. The variant of TTSR is used to develop NPD with more emphasis on the denoising problem. Figure 1 (below) shows the overall architecture of the NPD. It was built on top of the TTSR framework to adjust specifically to the denoising problem of continuous PPG signals.

NPD takes four inputs: (i) a noisy PPG signal, which is a (potential) mixture of noise and genuine PPG signals, (ii) a reference PPG signal, (iii) an upsampled signal of a downsampled reference PPG signal, and (iv) an upsampled signal of a noisy PPG signal. After extracting the learnable texture information from the reference PPG signal and a set of synthesized PPG signals (e.g., upsampled after downsampling a reference PPG signal, upsampled PPG signal), a transformer-based neural network learns the dominant feature information from the relation between them through the self-attention mechanism. Subsequently, the restored PPG signal is generated by considering both the noisy PPG signal and the attention vector learned from the reference PPG signal and a set of synthesized PPG signals.

## 3. Experimental Setup

Experiments were conducted to evaluate the performance of the NPD in various types of noise. The Mendeley data [14] and BIDMC [15] open datasets were used for this. Mendeley data were collected from healthy people with no control conditions, whereas the BIDMC was acquired from critically ill patients during hospital care at Beth Israel Deaconess Medical Center (Boston, MA, USA). The Mendeley dataset contains 2074 PPG signals obtained from 35 people. Each signal had a length of 6 s, and a sampling rate of 50 Hz. In the BIDMC dataset, 53 PPG signals were obtained from the 53 participants. Each signal had a length of 8 min, and a sampling rate of 125 Hz.

We resampled all PPG signals in both the datasets at 50 Hz, and thereafter segmented all the resampled signals to a length of 6 s. This was followed by normalization, each of which ranged between −1 and 1. Consequently, 6208 PPG signals were acquired. The entire resampled 6208 dataset was split into 2849 input data, 2849 reference data, and 510 test data for model training. Here, the input data referred to a noisy PPG signal, in which noise was added to the ground truth PPG signal.

**Figure 2:**
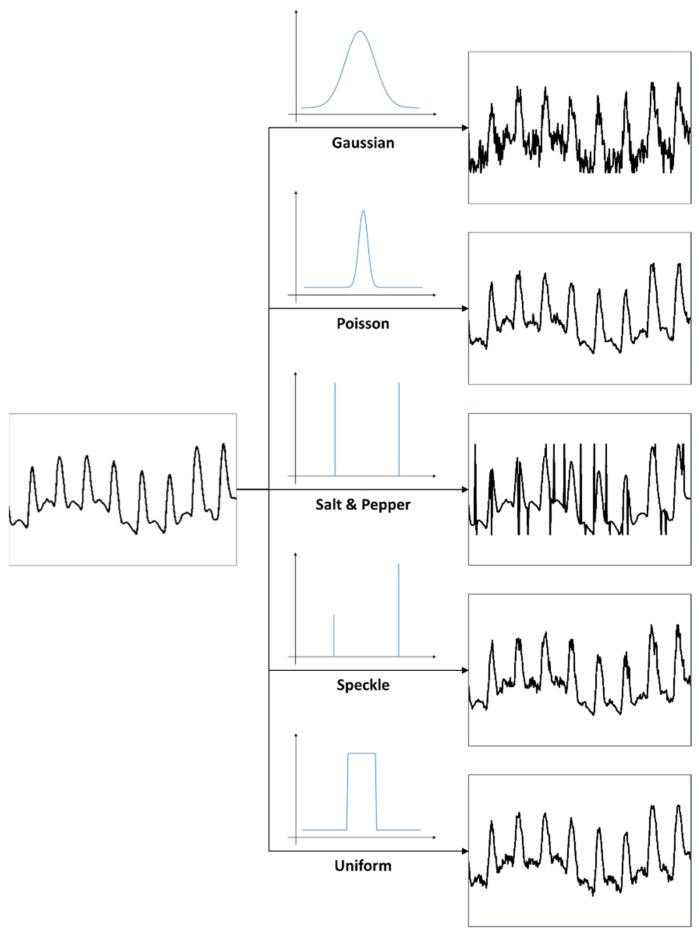
Example of the way in which a contaminated PPG signal was generated by adding noise to the original PPG signal.

For proper evaluation, a set of noisy PPG signals was synthetically generated from the obtained dataset. Next, the artificially contaminated PPG signals were denoised, and their denoising capacity examined, in comparison to the original PPG signals. Five different noises were added to the original dataset to compile the contaminated signals, as shown in Figure 2 (below): Gaussian noise, Poisson noise, salt-and-pepper noise, speckle noise, and uniform noise. The Gaussian noise and speckle noise follow a normal distribution, with a mean of 0 and a standard deviation of 0.01. Salt-and-pepper noise synthesizes only 5% of the ground truth, and uniform noise was sampled uniformly from 0 to 0.1. After synthesizing the normalized ground truth with noise, the signal was cropped to the range of 0–1. The synthesized signal was normalized to the range of −1 and 1.

## 4. Results

The mean squared error (MSE) and PSNR [16] were used for the evaluation of NPD. PSNR is the maximum signal-to-noise ratio computed by dividing the square of the maximum value of the signal by the MSE. Because all signals were normalized during the preprocessing stage, the maximum values of all signals were the same. Therefore, the PSNR was calculated directly from the MSE. The PSNR is defined as Equation (1):

(1) PSNR=−10log(MSE).

### 4.1. Case 1: Denoising a Single Noise

The NPD performance in denoising PPG signals was evaluated and compared with representative noise removal algorithms; here, a wavelet-based method [17] was chosen for the universal denoiser control condition. Additionally, the best algorithm for each type of noise was deployed. We expected that a performance comparison between NPD and the best model would clarify the competency of NPD. In other words, the study examined how a universal denoising approach performed compared to that of a specifically designed denoising algorithm.

For one specific noisy PPG signal, noise removal was conducted using three denoisers: NPD, wavelet-based method [17], and the best algorithms. The following filters were used when conducting a wavelet-based method:

(i). A discrete Meyer filter with decomposition level 3 for Gaussian noise.
(ii). A Daubechies 19 filter with decomposition level 3 for Poisson noise.
(iii). A Daubechies 18 filter with decomposition level 6 for salt-and-pepper noise.
(iv). A Daubechies 20 filter with decomposition level 3 for speckle noise.
(v). A Symlet 15 filter with a decomposition level of 9 for uniform noise.

For the best respective models for each noise type, the following [18,19] were used:

(i). A wiener filter with a window size of 5 for Gaussian noise.
(ii). A median filter with a window size of 3 for Poisson noise.
(iii). A median filter with a window size of 5 for salt-and-pepper noise.
(iv). A Weiner filter with a window size of 3 for speckle noise.
(v). A Gaussian filter with a sigma of 1 for uniform noise.

Following these settings, each denoiser’s PSNR was computed using the reference PPG signal and the denoised PPG signal.

**Figure 3:**
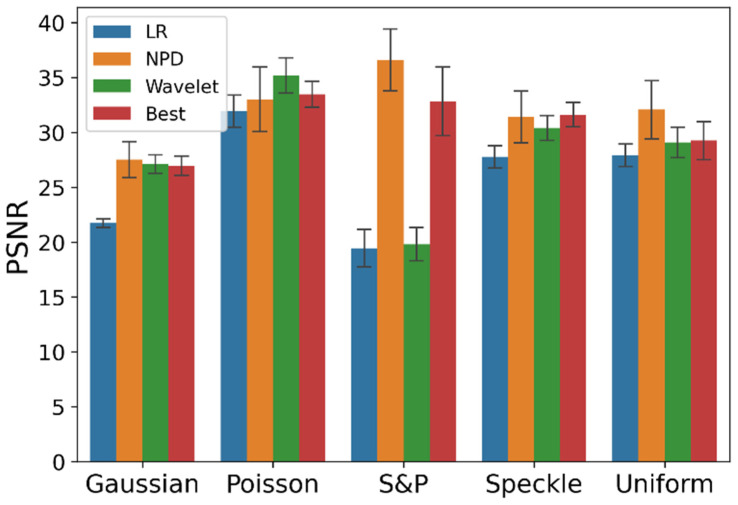
PSNR plot by single noise type and the denoising algorithm. The error bar is the standard deviation.

**Figure 4:**
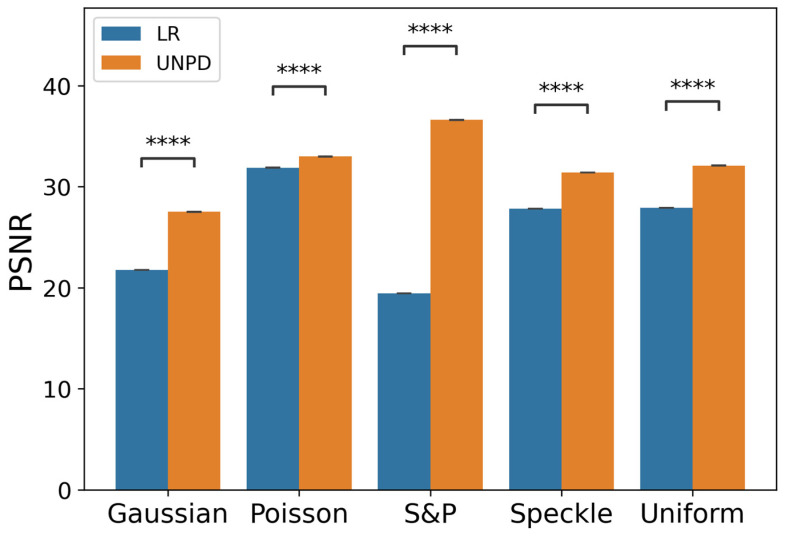
PSNR plot by single noise type with *p*-value from *t*-test (*N* = 50. ****: *p* < 1 × 10^−4^; paired-sample *t*-test. Error bars are the standard error of the mean (SEM)).

Table 1, and Figure 3 and Figure 4 show the evaluation results for a single noise. In Gaussian, salt and pepper, and uniform noise, NPD outperformed other denoising algorithms. In Poisson noise, NPD exhibited a lower performance than the other denoising algorithms. In speckle noise, NPD showed better performance than wavelet-based methods, but worse than the best respective algorithms. Although NPD takes a universal approach, it surpassed the performance of algorithms specifically designed for the distinctive characteristics of each noise, and performed at a similar level, even if it did not perform as well as these algorithms. 

### 4.2. Case 2: Denoising a Mixed Noise

**Figure 5:**
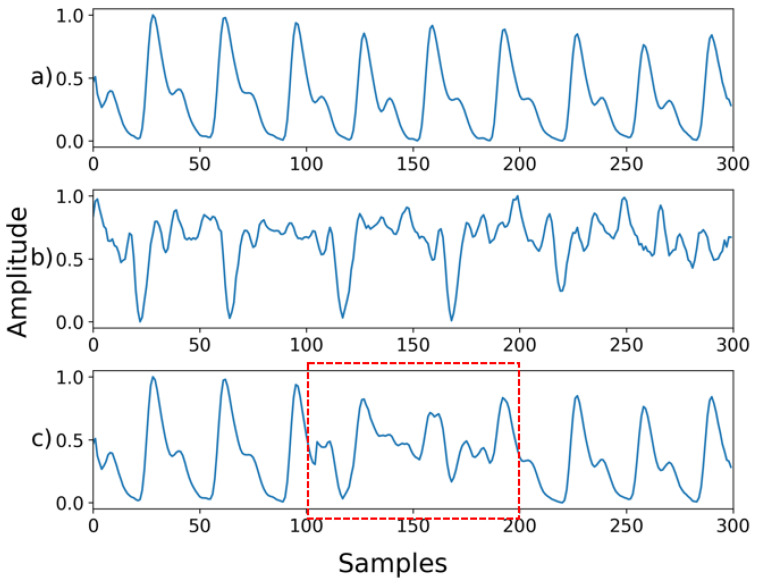
(**a**) Ground truth; (**b**) Motion artifact signal; (**c**) Synthesized signal with motion artifact added to 30% of the signal. The red box is the part that is synthesized with the motion artifact.

Next, we examined NPD’s capacity for denoising signals compromised by the mixture of multiple noises. In this case, the signal synthesized with two or more noises and that synthesized with motion artifacts were used as inputs. Mixed noise was generated by blending the noise shown in Figure 2. The type or order of the mixing noise is randomly determined. Motion artifacts were synthesized into the ground truth in the same manner as in Figure 5. In the motion artifact signal, the noise level is determined by the percentage of the total length of the ground truth synthesized by the motion artifact. The signal used for motion artifact synthesis was the wrist PPG dataset [20], which contains wrist PPGs recorded during walking, running, and bicycle riding. In the wrist PPG dataset, 19 PPG signals were obtained from eight participants. Each signal is a length of 10 min, and the sampling rate was 256 Hz. The motion artifact signal used for synthesis was used after resampling and normalization in the same manner as the ground truth signal.

**Figure 6:**
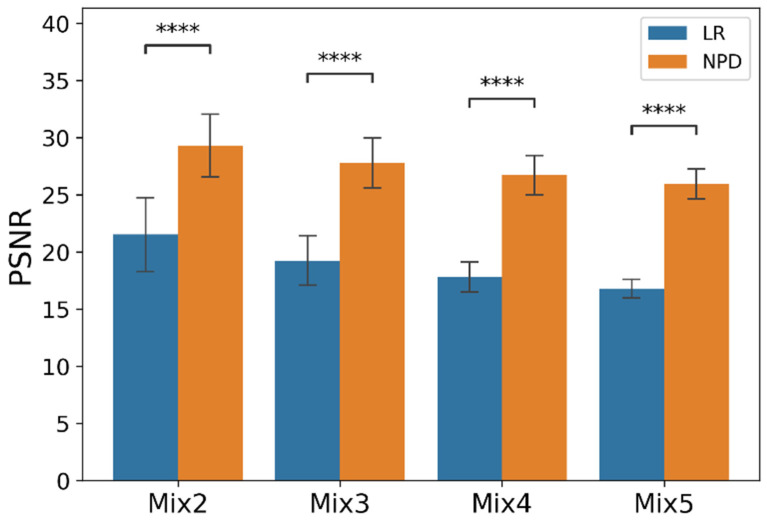
PSNR plot by mix noise type with *p*-value from *t*-test. (*N* = 50. ****: *p* < 1 × 10^−4^; paired-sample *t*-test. Error bars are the standard deviation).

Table 2 and Figure 6 (below) show the evaluation results for the mixed noise. As seen in Figure 6, the more noise was mixed, the smaller the deviation of PSNR because the deviation due to the type of noise became smaller. Further, as more noise was mixed, the PSNR of the synthesized signal and the PSNR of the recovered signal decreased together. It was also observed that the NPD was effective for mixed noise. 

### 4.3. Case 3: Denoising a Mixed Noise with Motion Artifacts

**Figure 7:**
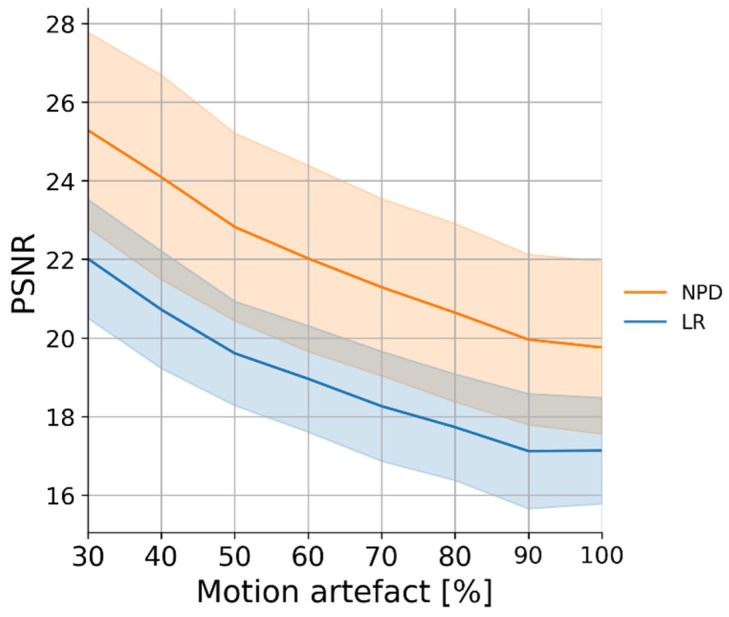
PSNR plot by motion artifact type.

**Figure 8:**
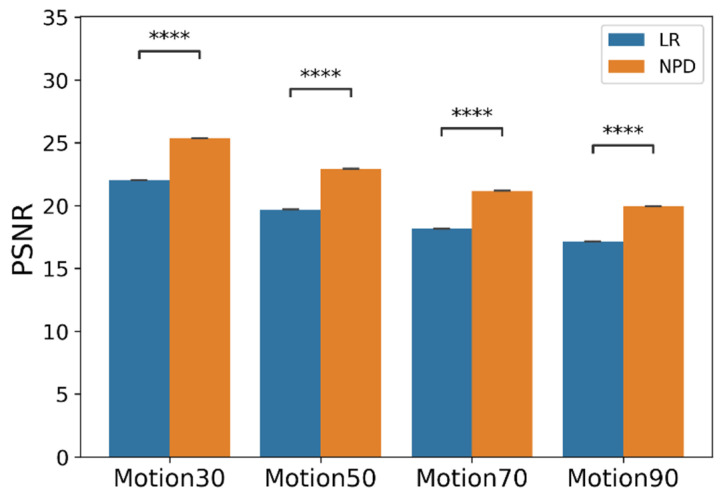
PSNR plot by motion artifact type with *p*-value from *t*-test. (*N* = 50. ****: *p* < 1 × 10^−4^; paired-sample *t*-test. Error bars are the SEM).

We further conducted the experiment on the mixed noise case with motion artifacts. Table 3 and Figure 7 and Figure 8 (below) show the evaluation results for this. Figure 7 shows the mean and standard deviation of the PSNR. When the motion noise ratio was less than 30%, the signal change was not large; thus, the result was not obtained. The recovery result of a signal synthesized with motion artifacts showed the worst performance compared to other noises. However, it also showed that the signal can be recovered by removing the motion artifact at all motion artifact synthesis ratios, even at 100%. 

### 4.4. Validation of the NPD model

#### 4.4.1. NPD’s Denoising Capacity across the Sampling Rate of Inputs Signals 

The NPD used for the whole experiments illustrated above was trained with input signals resampled according to Mendeley data [14]. However, similar to BIDMC data [15] and wrist PPG data [20], there are data of various sampling rates, such as 125 Hz and 256 Hz, and the PPG signal obtained by a more precise machine has a 1 kHz sampling rate. To observe the results according to these various sampling rates, the PSNR results were obtained by changing only the sampling rate of the input signal while maintaining the NPD model. Experimental results are shown in Table 4 below; even though NPD learned by fixing the sampling rate of the input signal to 50 Hz, it removed single noise and mixed noise of 1 kHz and 125 Hz input signals well, and some noises showed better results than 50 Hz. However, the motion artifact was not well removed, and 1 kHz was hardly removed. This appears to be caused by the difference between the sampling rate of the signal used for learning and the sampling rate of the input signal. If the sampling rate of the input signal of the NPD is set according to the data and learned, it is expected to show performance even in an environment using 1 kHz data.

#### 4.4.2. NPD’s Denoising Capacity to the Real World PPG Data 

**Figure 9:**
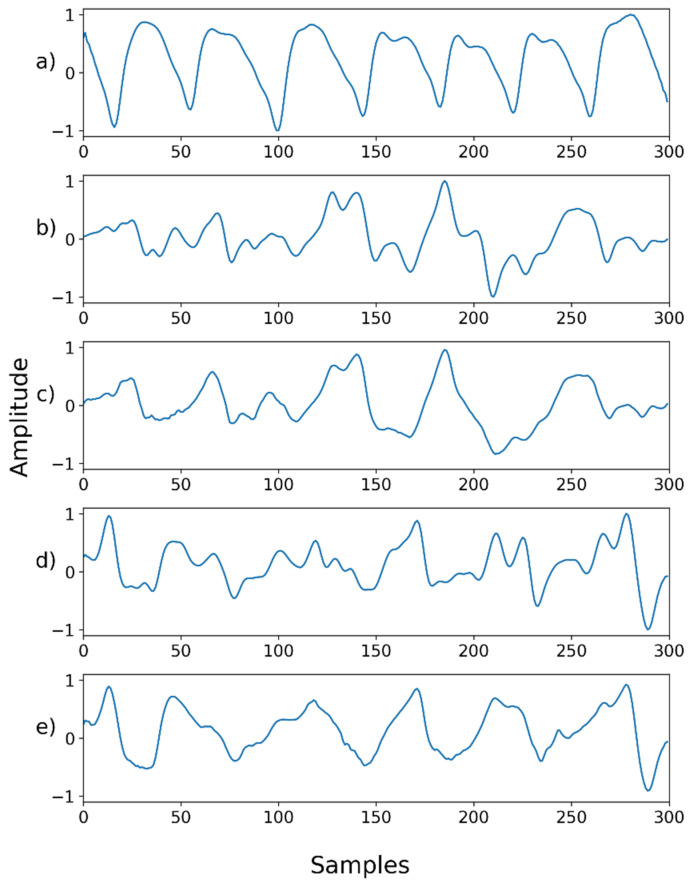
(**a**) Reference signal; (**b**) real raw PPG signal 1; (**c**) restored b by NPD; (**d**) real raw PPG signal 2; (**e**) restored d by NPD.

We additionally examined the NPD’s robust denoising capacity using real raw PPG signals and nonsynthesized signals, which we never used for the NPD training. The dataset used was the PPG-DaLia dataset [21], the sampling rate of the PPG signal was 64 Hz, and the signals were obtained from 15 people. The PPG-DaLia dataset consists of a clean PPG signal obtained from a sitting state and a PPG signal obtained from seven activities, such as cycling, walking, and going up and down the stairs. Figure 9 shows the real raw PPG signal and the signal recovered by the NPD. A clean PPG signal obtained from the PPG-DaLia dataset was used as the reference signal, and a signal with motion artifacts was used as the input. Although a performance evaluation is required, the PSNR value cannot be obtained because the real raw PPG signal does not have the original signal from which noise has been removed. Therefore, the PSNR value obtained by comparison with the reference signal was used. We performed a *t*-test to see whether the average of the difference between the PSNR of the real raw PPG signal and the PSNR of the signal recovered by NPD was 0. The signal used in the *t*-test was a reference signal and a real raw PPG signal obtained by repeating sampling 100 times from the PPG signal of the same person. As a result of the *t*-test, the *p*-value is less than 0.0001; therefore, it can be seen that the real raw PPG signal and the signal recovered with NPD are statistically different signals. However, we note that it is not known whether the signal recovered by NPD is correctly recovered.

#### 4.4.3. NPD’s Sensitivity in Denoising 

**Figure 10:**
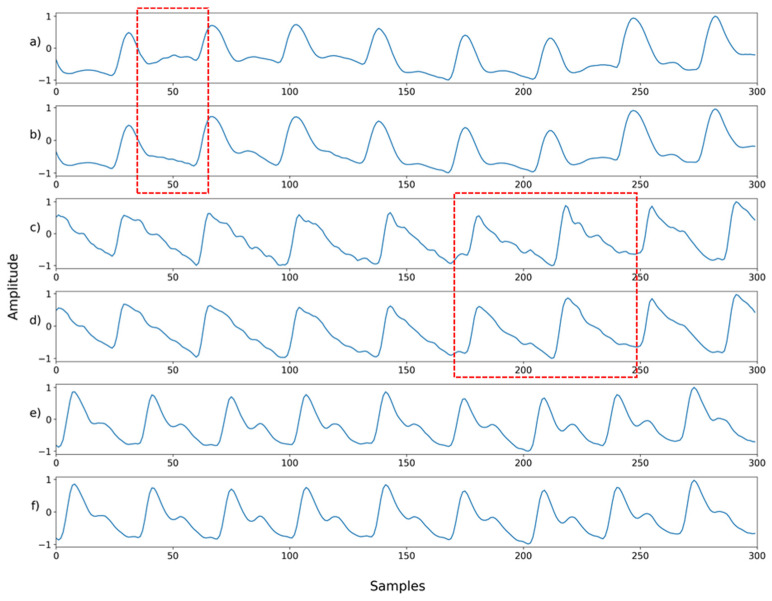
(**a**) Sample 0′s ground truth; (**b**) restored a by NPD; (**c**) sample 1′s ground truth; (**d**) restored c by NPD; (**e**) sample 2′s ground truth; (**f**) restored e by NPD. The red box where the ground truth was recognized as noise. Restoring the original signal with NPD shows that the signal is distorted.

Next, we designed another experiment to validate the NPD’s sensitivity—the NPD would not be able to distort the signal if there is no noise imposed in the input signal (e.g., a ground truth signal). To find out the signal distortion by the NPD, an experiment was conducted with the ground truth as input. As a result, the average PSNR was 39.2526, which is fairly large compared to other noises; however, it was confirmed that distortion occurred. Figure 10 (below) shows the part of the ground truth that is incorrectly recognized as noise. The part incorrectly recognized as noise becomes smoother than the input signal, and the pattern changes. These parts generally contain high-frequency information or have a shape similar to that of a motion artifact. As shown in (e) in Figure 10, if there is no high-frequency information and there is no similarity to motion noise, almost the same signal as the input is output, even if it passes the NPD.

#### 4.4.4. NPD’s Denoising Results 

**Figure 11:**
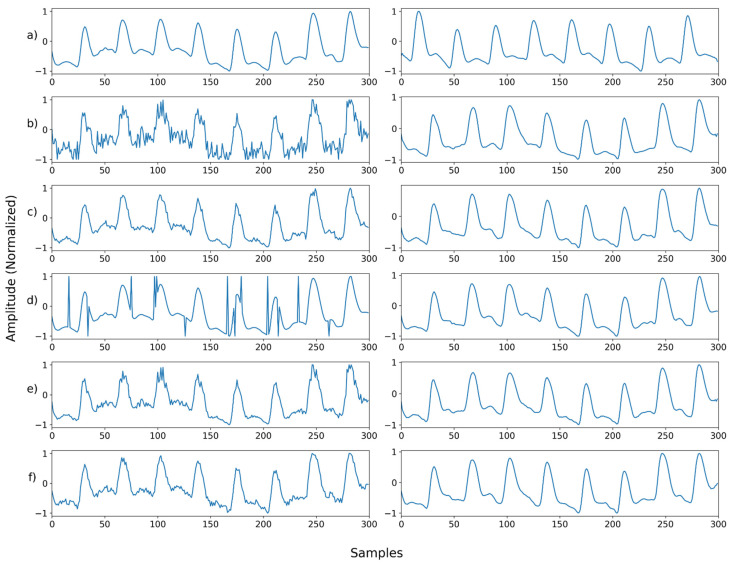
Single noise—Example 1: (**a**) Left: ground truth, Right: reference signal; (**b**) Left: synthesized signal with Gaussian noise, Right: restored left signal by NPD; (**c**) Left: synthesized signal with Poisson noise, Right: restored left signal by NPD; (**d**) Left: synthesized signal with salt-and-pepper noise, Right: restored left signal by NPD; (**e**) Left: synthesized signal with speckle noise, Right: restored left signal by NPD; (**f**) Left: synthesized signal with uniform noise, Right: restored left signal by NPD.

**Figure 12:**
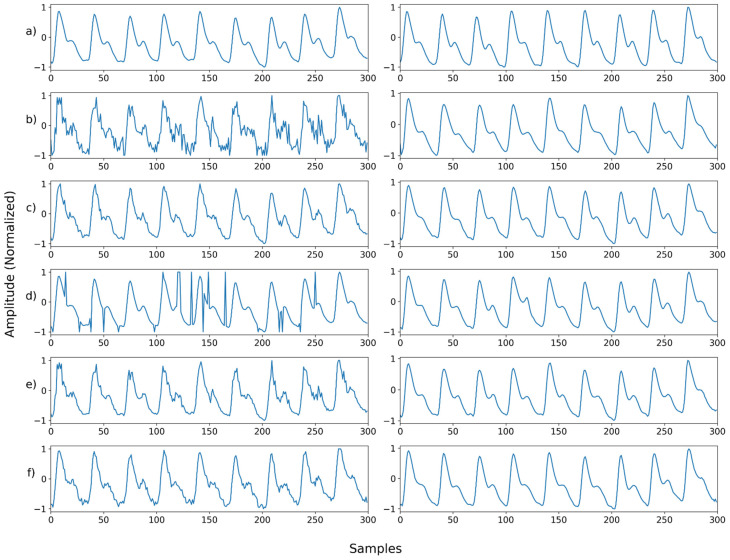
Single noise—Example 2: (**a**) Left: ground truth, Right: reference signal; (**b**) Left: synthesized signal with Gaussian noise, Right: restored left signal by NPD; (**c**) Left: synthesized signal with Poisson noise, Right: restored left signal by NPD; (**d**) Left: synthesized signal with salt-and-pepper noise, Right: restored left signal by NPD; (**e**) Left: synthesized signal with speckle noise, Right: restored left signal by NPD; (**f**) Left: synthesized signal with uniform noise, Right: restored left signal by NPD.

**Figure 13:**
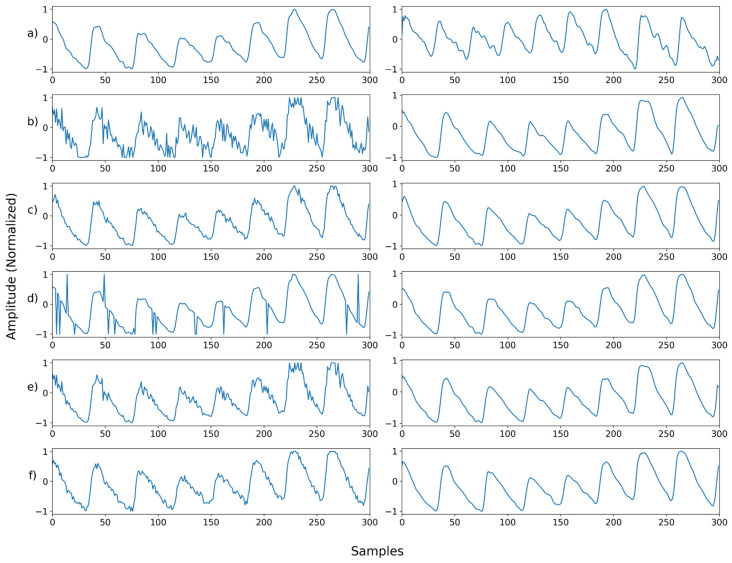
Single noise—Example 3: (**a**) Left: ground truth, Right: reference signal; (**b**) Left: synthesized signal with Gaussian noise, Right: restored left signal by NPD; (**c**) Left: synthesized signal with Poisson noise, Right: restored left signal by NPD; (**d**) Left: synthesized signal with salt-and-Pepper noise, Right: restored left signal by NPD; (**e**) Left: synthesized signal with speckle noise, Right: Restored left signal by NPD; (**f**) Left: synthesized signal with uniform noise, Right: restored left signal by NPD.

**Figure 14:**
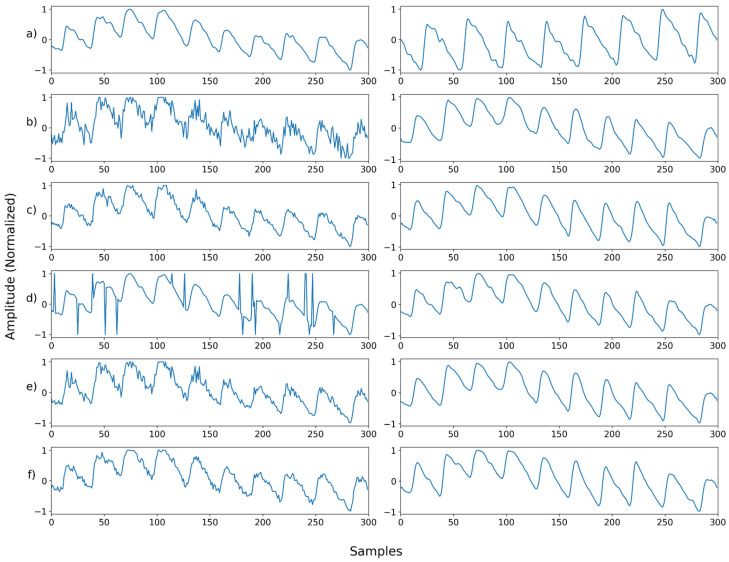
Single noise—Example 4: (**a**) Left: ground truth, Right: reference signal; (**b**) Left: synthesized signal with Gaussian noise, Right: restored left signal by NPD; (**c**) Left: synthesized signal with Poisson noise, Right: restored left signal by NPD; (**d**) Left: synthesized signal with salt-and-pepper noise, Right: restored left signal by NPD; (**e**) Left: synthesized signal with speckle noise, Right: restored left signal by NPD; (**f**) Left: synthesized signal with uniform noise, Right: restored left signal by NPD.

**Figure 15:**
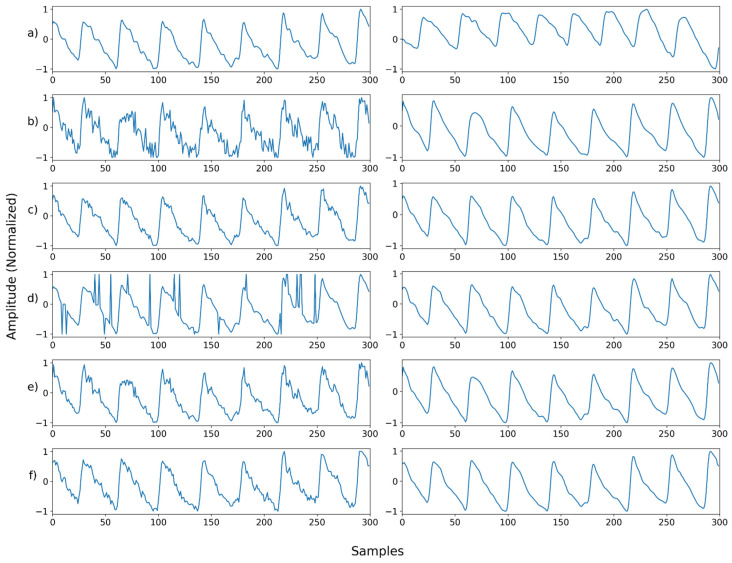
Single noise—Example 5: (**a**) Left: ground truth, Right: reference signal; (**b**) Left: synthesized signal with Gaussian noise, Right: restored left signal by NPD; (**c**) Left: synthesized signal with Poisson noise, Right: restored left signal by NPD; (**d**) Left: synthesized signal with salt-and-pepper noise, Right: restored left signal by NPD; (**e**) Left: synthesized signal with speckle noise, Right: restored left signal by NPD; (**f**) Left: synthesized signal with uniform noise, Right: restored left signal by NPD.

**Figure 16:**
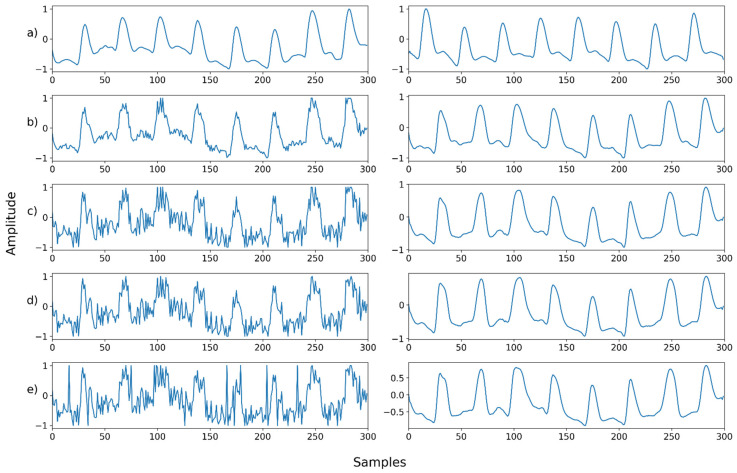
Mix noise—Example 1: (**a**) Left: ground truth, Right: reference signal; (**b**) Left: synthesized signal with speckle noise and uniform noise, Right: restored left signal by NPD; (**c**) Left: synthesized signal with b and Gaussian noise, Right: restored left signal by NPD; (**d**) Left: synthesized signal with c and Poisson noise, Right: restored left signal by NPD; (**e**) Left: synthesized signal with d and salt-and-pepper noise, Right: restored left signal by NPD.

**Figure 17:**
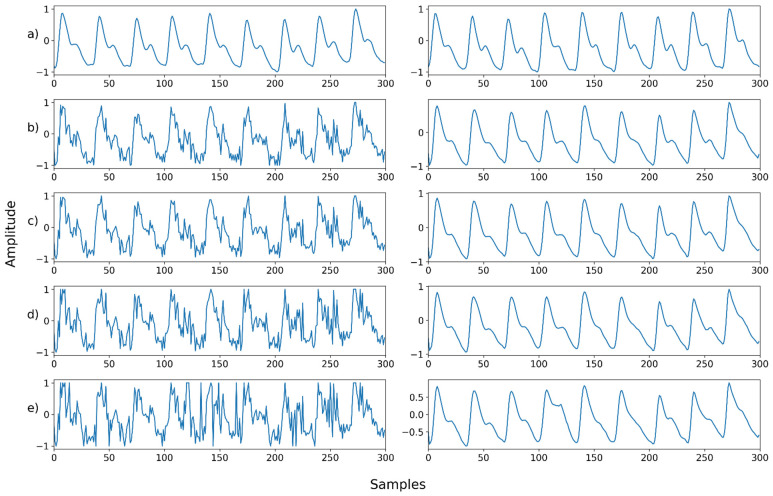
Mix noise—Example 2: (**a**) Left: ground truth, Right: reference signal; (**b**) Left: synthesized signal with Poisson noise and Gaussian noise, Right: restored left signal by NPD; (**c**) Left: synthesized signal with b and uniform noise, Right: restored left signal by NPD; (**d**) Left: synthesized signal with c and speckle noise, Right: restored left signal by NPD; (**e**) Left: synthesized signal with d and salt-and-pepper noise, Right: restored left signal by NPD.

**Figure 18:**
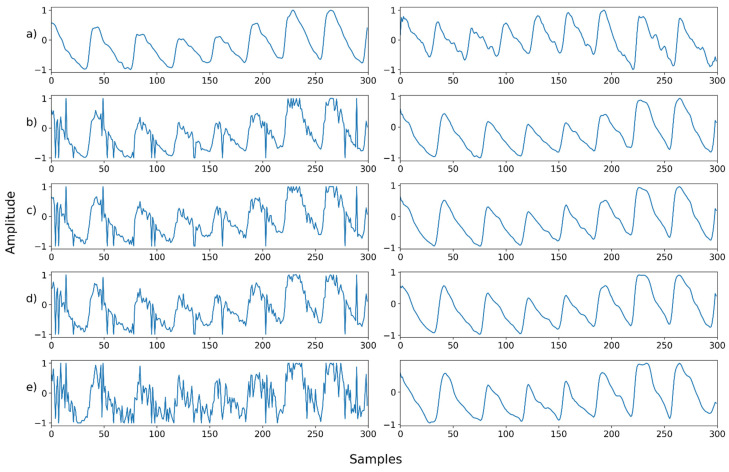
Mix noise—Example 3: (**a**) Left: ground truth, Right: reference signal; (**b**) Left: synthesized signal with speckle noise and salt-and-pepper noise, Right: restored left signal by NPD; (**c**) Left: synthesized signal with b and uniform noise, Right: restored left signal by NPD; (**d**) Left: synthesized signal with c and Poisson noise, Right: restored left signal by NPD; (**e**) Left: synthesized signal with d and Gaussian noise, Right: restored left signal by NPD.

**Figure 19:**
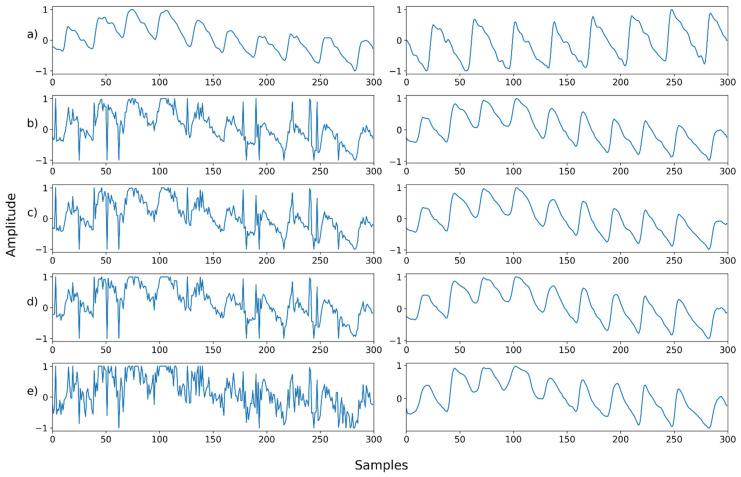
Mix noise—Example 4: (**a**) Left: ground truth, Right: reference signal; (**b**) Left: synthesized signal with salt-and-pepper noise and speckle noise, Right: restored left signal by NPD; **(c**) Left: synthesized signal with b and Poisson noise, Right: restored left signal by NPD; (**d**) Left: synthesized signal with c and uniform noise, Right: restored left signal by NPD; (**e**) Left: synthesized signal with d and Gaussian noise, Right: restored left signal by NP.

**Figure 20:**
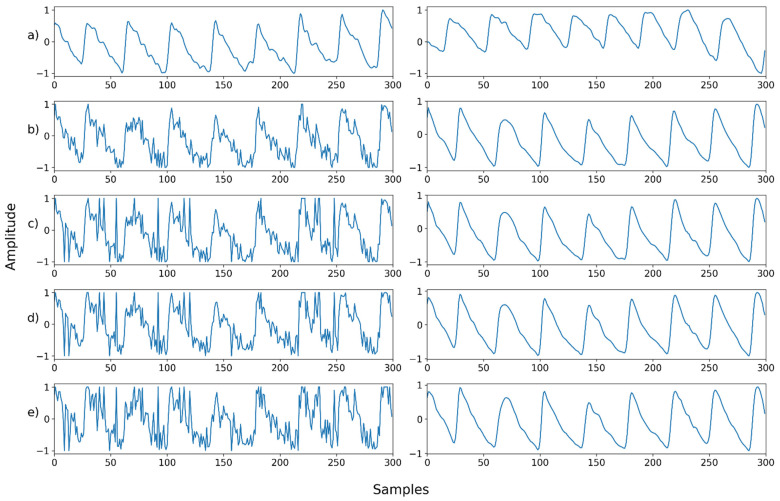
Mix noise—Example 5: (**a**) Left: ground truth, Right: reference signal; (**b**) Left: synthesized signal with Poisson noise and Gaussian noise, Right: restored left signal by NPD; (**c**) Left: synthesized signal with b and salt-and-pepper noise, Right: restored left signal by NPD; (**d**) Left: synthesized signal with c and uniform noise, Right: restored left signal by NPD; (**e**) Left: synthesized signal with d and speckle noise, Right: restored left signal by NPD.

**Figure 21:**
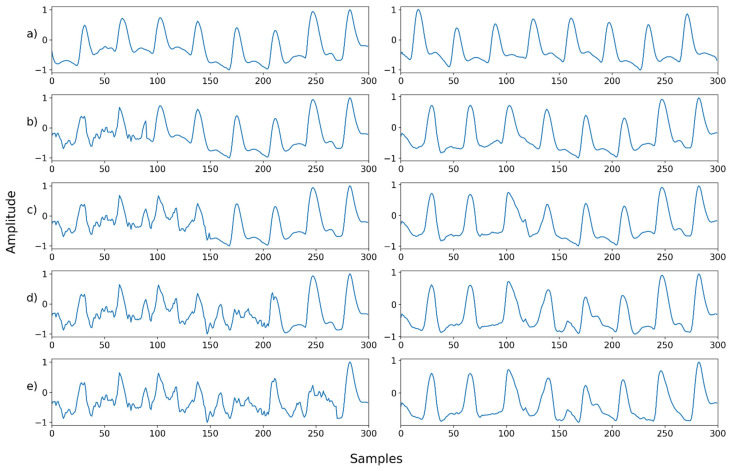
Motion noise—Example 1: (**a**) Left: ground truth, Right: reference signal; (**b**) Left: synthesized signal with motion artifact added to 30% of the signal, Right: restored left signal by NPD; (**c**) Left: synthesized signal with motion artifact added to 50% of the signal, Right: restored left signal by NPD; (**d**) Left: synthesized signal with motion artifact added to 70% of the signal, Right: restored left signal by NPD; (**e**) Left: synthesized signal with motion artifact added to 90% of the signal, Right: restored left signal by NPD. The starting point of motion artifact synthesis is the starting point of the signal.

**Figure 22:**
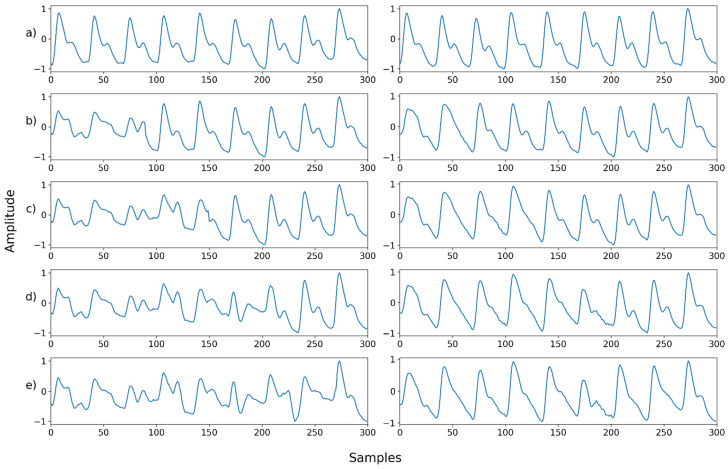
Motion noise—Example 2: (**a**) Left: ground truth, Right: reference signal; (**b**) Left: synthesized signal with motion artifact added to 30% of the signal, Right: restored left signal by NPD; (**c**) Left: synthesized signal with motion artifact added to 50% of the signal, Right: restored left signal by NPD; (**d**) Left: synthesized signal with motion artifact added to 70% of the signal, Right: restored left signal by NPD; (**e**) Left: synthesized signal with motion artifact added to 90% of the signal, Right: restored left signal by NPD. The starting point of motion artifact synthesis is the starting point of the signal.

**Figure 23:**
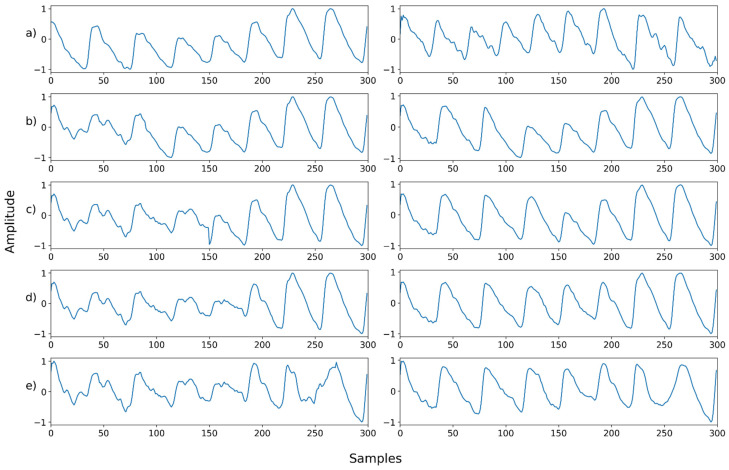
Motion noise—Example 3: (**a**) Left: ground truth, Right: reference signal; (**b**) Left: synthesized signal with motion artifact added to 30% of the signal, Right: restored left signal by NPD; (**c**) Left: synthesized signal with motion artifact added to 50% of the signal, Right: restored left signal by NPD; (**d**) Left: synthesized signal with motion artifact added to 70% of the signal, Right: restored left signal by NPD; (**e**) Left: synthesized signal with motion artifact added to 90% of the signal, Right: restored left signal by NPD. The starting point of motion artifact synthesis is the starting point of the signal.

**Figure 24:**
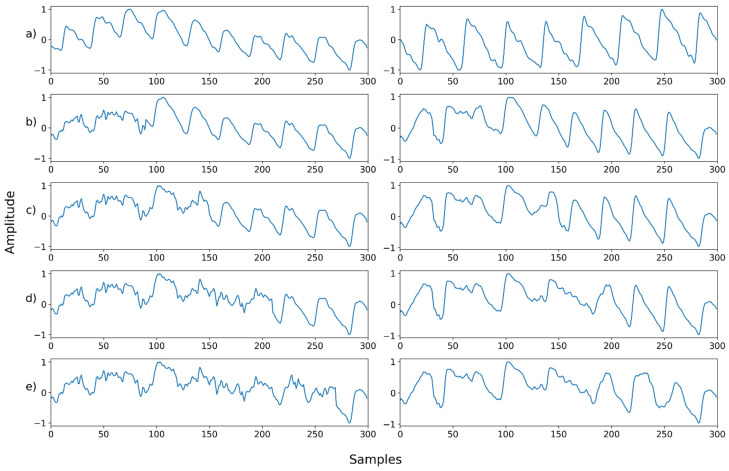
Motion noise—Example 4: (**a**) Left: ground truth, Right: reference signal; (**b**) Left: synthesized signal with motion artifact added to 30% of the signal, Right: restored left signal by NPD; (**c**) Left: synthesized signal with motion artifact added to 50% of the signal, Right: restored left signal by NPD; (**d**) Left: synthesized signal with motion artifact added to 70% of the signal, Right: restored left signal by NPD; (**e**) Left: synthesized signal with motion artifact added to 90% of the signal, Right: restored left signal by NPD. The starting point of motion artifact synthesis is the starting point of the signal.

**Figure 25:**
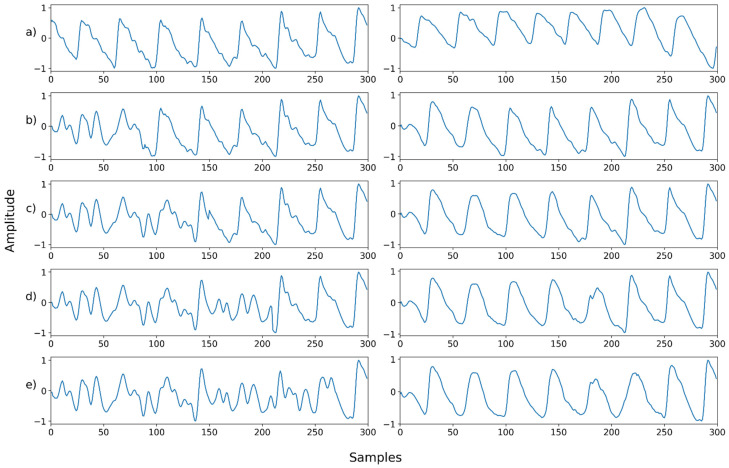
Motion noise—Example 5: (**a**) Left: ground truth, Right: reference signal; (**b**) Left: synthesized signal with motion artifact added to 30% of the signal, Right: restored left signal by NPD; (**c**) Left: synthesized signal with motion artifact added to 50% of the signal, Right: restored left signal by NPD; (**d**) Left: synthesized signal with motion artifact added to 70% of the signal, Right: restored left signal by NPD; (**e**) Left: synthesized signal with motion artifact added to 90% of the signal, Right: restored left signal by NPD. The starting point of motion artifact synthesis is the starting point of the signal.

Figure 11, Figure 12, Figure 13, Figure 14, Figure 15, Figure 16, Figure 17, Figure 18, Figure 19, Figure 20, Figure 21, Figure 22, Figure 23, Figure 24 and Figure 25 show a snapshot of the ground truth PPG signal (i.e., the PPG signal before noise is added to it), the reference PPG signal used for training of NPD, and examples of PPG signals: one is the input PPG signal in which noise is added to the ground truth PPG signal (left column) and the other is the PPG signal restored by NPD (right column). Five fixed PPG signals were used for each noise, mixed noise, and motion artifact type. In the case of the motion artifact type, the motion artifact was synthesized from the beginning of the signal for comparison of results, but in reality, it is possible to recover from wherever it is synthesized. Given the PSNR results in Table 1, Table 2 and Table 3, all types of noise were removed, and, thus, all PPG signals were restored.

## 5. Conclusions

This study proposes a universal computational framework for denoising PPG signals, which the authors have named NPD. By casting the denoising problem into an image-restoration problem using a SR approach, the authors were able to build transformer-based deep generative models that could facilitate the universal noise removal of continuous PPG signals using a variant of an image-based TTSR framework [12]. Open datasets Mendeley data [14] and BIDMC [15] were used for NPD learning and performance evaluation. The PPG signals obtained from the dataset were resampled to 50 Hz and normalized to a range of −1 to 1. For the performance evaluation, a signal synthesized with a single noise, mixed noise, and motion artifact was used. Gaussian noise, Poisson noise, salt-and-pepper noise, speckle noise, and uniform noise were included in this study. Mix noise is noise that is randomly selected from among single noises and mixed in a random order. For the motion artifact, the signal obtained from the wrist PPG dataset [20] was used. As a result of single noise restoration, Gaussian noise, salt-and-pepper noise, and uniform noise showed better results than the wavelet-based method or the best respective algorithm, and their respective PSNRs were 27.5084, 36.6080, and 32.0615. For Poisson noise and speckle noise, the PSNR was 33.0090 and 31.4139, respectively; therefore, the performance was poor compared to other methods, but noise was removed to a similar level. For the mixed noise, the experiment was carried out by increasing the number of mixed noises from two to five. The PSNR values were 29.2759, 27.8759, 26.5608, and 25.9402, which removed the mixed noise well in all the cases. The synthesized motion artifact signal was created by synthesizing only a certain ratio of the total signal length. As a result of the motion artifact signal restoration, the PSNRs were 25.2872, 22.8240, 21.2901, and 19.9577 at 30%, 50%, 70%, and 90% motion artifact ratios, respectively. Although the results of the recovery of motion artifact signals were not as good as those of other noises, it can be confirmed that NPD can also remove motion artifacts.

The findings on this study open optimistic possibilities for designing a universal denoiser for continuous PPG signals. The authors believe that NPD can provide a universal and robust means to eliminate the known and/or unknown noise in PPG signals in any type of noise. In other words, this solution is potentially “one-for-all” in the PPG processing domain. This could significantly contribute to reducing the time and effort required to design denoising algorithms for a specific type of noise.

Future work will focus on experiments that use real-world PPG signals, given that this study only dealt with synthesized and manipulated PPG signals using open datasets. New experiments may also be investigated in the future, focused on continuous PPG signals in which multiple noises are simultaneously added to the PPG signals so that a single denoising algorithm or cascade connection of denoising algorithms cannot be resolved.

## Figures and Tables

**Table 1:** Experimental results for single noise.

Noise Type	Noisy Signals		Denoised Signals					
			NPD		Wavelet-Based		Best Respective Algorithm	
	PSNR	MSE	PSNR	MSE	PSNR	MSE	PSNR	MSE
Gaussian	21.7293	0.0067	27.5084	0.0019	27.1040	0.0019	26.9657	0.0020
Poisson	31.9316	0.0006	33.0090	0.0007	35.1822	0.0003	33.4795	0.0004
Salt and Pepper	19.4427	0.0121	36.6080	0.0002	19.8160	0.0110	32.8234	0.0006
Speckle	27.7579	0.0017	31.4139	0.0008	30.4084	0.0009	31.6180	0.0007
Uniform	27.9224	0.0016	32.0615	0.0007	29.0833	0.0012	29.2538	0.0012

**Table 2:** Experimental results for mix noise.

Noise Type	Noisy Signals		NPD	
	PSNR	MSE	PSNR	MSE
Mix 2 Noise	21.5033	0.0089	29.2759	0.0014
Mix 3 Noise	19.1682	0.0134	27.8759	0.0018
Mix 4 Noise	17.8539	0.0172	26.5608	0.0023
Mix 5 Noise	16.8180	0.0211	25.9402	0.0026

**Table 3:** Experimental results for motion artifact.

Noise Type	Noisy Signals		NPD	
	PSNR	MSE	PSNR	MSE
Motion Artifact (30%)	22.0146	0.0066	25.2872	0.0034
Motion Artifact (50%)	19.6071	0.0114	22.8240	0.0060
Motion Artifact (70%)	18.2607	0.0157	21.2901	0.0085
Motion Artifact (90%)	17.1186	0.0205	19.9577	0.0115

**Table 4:** Experimental results according to sampling rate of the input signal.

Noise Type	Noisy Signals			NPD		
	PSNR			PSNR		
Sampling Rate (Hz)	1k	125	50	1k	125	50
Gaussian	21.7103	21.7176	21.7293	26.0019	27.2057	27.5084
Poisson	44.0201	35.1580	31.9316	37.3230	33.5451	33.0090
Salt and Pepper	19.2218	19.3412	19.4427	38.4069	38.8832	36.6080
Speckle	27.7810	27.8330	27.7579	29.9358	31.1394	31.4139
Uniform	26.6985	27.3312	27.9224	29.0393	31.1699	32.0615
Mix 2	21.5791	21.5683	21.5033	28.6071	29.2880	29.2759
Mix 3	19.2486	19.2116	19.1682	27.1239	27.7867	27.8759
Mix 4	17.7821	17.8783	17.8539	26.1827	26.7947	26.5608
Mix 5	16.8197	16.7760	16.8180	25.3941	26.0695	25.9402
Motion Artifact (30%)	22.2445	22.0266	22.0146	22.1312	22.5699	25.2872
Motion Artifact (50%)	19.8380	19.8349	19.6071	19.7609	20.2669	22.8240
Motion Artifact (70%)	18.3859	18.2725	18.2607	18.3166	18.6561	21.2901
Motion Artifact (90%)	17.3457	17.3020	17.1186	17.2753	17.6354	19.9577

## Data Availability

The data presented in this study are openly available in Mendeley Data at 10.17632/yynb8t9x3d.1 (accessed on 5 March 2022), reference number [14], The data presented in this study are openly available in PhysioNet at https://doi.org/10.13026/C2208R (accessed on 5 March 2022), reference number [15]. Publicly available datasets were analyzed in this study. This data can be found here: https://archive.ics.uci.edu/ml/datasets/PPG-DaLiA (accessed on 5 March 2022).
